# LncRNA HOXA11‐AS regulates calcium oxalate crystal–induced renal inflammation via miR‐124‐3p/MCP‐1

**DOI:** 10.1111/jcmm.14706

**Published:** 2019-11-03

**Authors:** Yinhui Li, Guiling Yan, Jie Zhang, Wei Chen, Tao Ding, Yupeng Yin, Minghan Li, Yiqing Zhu, Shuhan Sun, Ji Hang Yuan, Zhiyong Guo

**Affiliations:** ^1^ Department of Nephrology Changhai Hospital The Naval Military Medical University Shanghai China; ^2^ Department of Breast and Thyroid Surgery Changhai Hospital The Naval Military Medical University Shanghai China; ^3^ Department of General Surgery The Naval Hospital Eastern Theater PLA Zhoushan Zhejiang China; ^4^ Department of Medical Genetics The Naval Military Medical University Shanghai China

**Keywords:** calcium oxalate, HOXA11‐AS, lncRNA, MCP‐1, mir‐124‐3p

## Abstract

Long noncoding RNA (lncRNA) has been suggested to play an important role in a variety of diseases over the past decade. In a previous study, we identified a novel lncRNA, termed HOXA11‐AS, which was significantly up‐regulated in calcium oxalate (CaOx) nephrolithiasis. However, the biological function of HOXA11‐AS in CaOx nephrolithiasis remains poorly defined. Here, we demonstrated that HOXA11‐AS was significantly up‐regulated in CaOx nephrolithiasis both in vivo and in vitro. Gain‐/loss‐of‐function studies revealed that HOXA11‐AS inhibited proliferation, promoted apoptosis and aggravated cellular damage in HK‐2 cells exposed to calcium oxalate monohydrate (COM). Further investigations showed that HOXA11‐AS regulated monocyte chemotactic protein 1 (MCP‐1) expression in HK‐2 cell model of CaOx nephrolithiasis. In addition, online bioinformatics analysis and dual‐luciferase reporter assay results showed that miR‐124‐3p directly bound to HOXA11‐AS and the 3'UTR of MCP‐1. Furthermore, rescue experiment results revealed that HOXA11‐AS functioned as a competing endogenous RNA to regulate MCP‐1 expression through sponging miR‐124‐3p and that overexpression of miR‐124‐3p restored the inhibitory effect of proliferation, promotion effects of apoptosis and cell damage induced by HOXA11‐AS overexpression. Taken together, HOXA11‐AS mediated CaOx crystal–induced renal inflammation via the miR‐124‐3p/MCP‐1 axis, and this outcome may provide a good potential therapeutic target for nephrolithiasis.

## 1 INTRODUCTION

Nephrolithiasis, also known as kidney stone, is one of the most common diseases of the urinary system with a lifetime prevalence of nearly 6% in women and 10% in men.^1^ It is also an important cause of chronic kidney disease (CKD) and end‐stage kidney disease (ESRD).^2^ The emergence of minimally invasive surgical technology has spurred great developments in the treatment of nephrolithiasis. However, a high recurrence rate of approximately 60% in ten years after the first treatment and high prevalence pose a serious economic burden to people worldwide.^1^ The main component of renal stones in more than 80% of patients is calcium oxalate (CaOx), which consists mainly of calcium oxalate monohydrate (COM) and calcium oxalate dihydrate (COD), with COM representing the major form.^1^ However, the therapeutic effect of nonoperative methods on the prevention and treatment of new or recurrent renal calculi is largely limited, and the pathogenesis of calcium oxalate stone is still not sufficiently clear. Therefore, the pathogenesis of calcium oxalate stones and the search for new therapeutic targets are urgently needed.

Numerous studies have shown that calcium oxalate stone formation is an inflammatory process.^3^, ^4^ The CaOx crystals in the kidney interstitium are always surrounded by numerous macrophages in specimens from stone patients and in animal models.^3^ When exposed to COM or oxalate (Ox), renal tubular epithelial cells secrete many inflammatory factors, such as osteopontin (OPN), monocyte chemotactic protein 1 (MCP‐1), CD44 and transforming growth factor‐β (TGF‐β), among others.^4^, ^5^ MCP‐1 is one of the well‐accepted and most important inflammatory factors in kidney stones. As a potent monocyte attractant, MCP‐1 has been reported to account for nearly 70%‐80% of the monocyte chemotactic activity in various types of renal inflammation.^3^ Monocytes are chemotactically attracted and converge to inflammatory sites where they are activated by COM and inflammatory factors and then secrete additional proinflammatory cytokines that attract more inflammatory cells, thereby aggravating the kidney damage via an amplification loop.^6^ Consequently, MCP‐1 plays a trigger‐point role in the inflammatory cascade of calcium oxalate stone formation.

Increasing evidence has shown that long noncoding RNAs (lncRNAs), which are members of the noncoding RNA family with a length exceeding 200 nucleotides, are involved in various cancerous and noncancerous diseases.^7^, ^8^, ^9^, ^10^ In a previous study, using a microarray technique and BLAST algorithm, we identified 15 lncRNA homologs in mice and humans with aberrant expression in glyoxylate‐induced calcium oxalate stone mouse kidneys and demonstrated that lncRNA CHCHD4P4 promoted epithelial‐mesenchymal transition (EMT) in CaOx nephrolithiasis.^8^ Recently, a novel lncRNA, namely, HOXA11‐AS on the list in our microarray reporter, has attracted great attention for its role in a variety of diseases. For instance, HOXA11‐AS has been demonstrated to promote gastric cancer tumorigenesis and progression by scaffolding the chromatin modification factors PRC2, LSD1 and DNMT1.^10^ In another study, HOXA11‐AS was found to be a tumour suppressor gene in epithelial ovarian cancer.^11^ In addition, HOXA11‐AS has been suggested to delay fracture healing via sponging miR‐124‐3p^12^ and to promote diabetic arteriosclerosis–related inflammation through the PI3K/AKT pathway.^13^ Taken together, these findings reveal that HOXA11‐AS functions in a tissue‐ and cell‐type‐specific manner like other lncRNAs.^14^ However, its biological function in calcium oxalate crystal–induced renal injury remains poorly defined. Therefore, the aim of this study was to investigate the function and underlying mechanism of HOXA11‐AS in CaOx renal stones.

## 2 MATERIALS AND METHODS

### 2.1 Animal studies and von Kossa staining

A total of 12 male C57BL/6 mice, aged 8 weeks and weighing approximately 18‐22 g, were obtained from the Shanghai SLAC Laboratory Animal Co., Ltd. The current protocols were approved by the Animal Care and Use Committee of the Second Military Medical University. CaOx crystals were induced by successive intraperitoneal injections of glyoxylate (G0366, TCI) at 120 mg/kg per mouse once daily for 2 weeks. After acclimatization for 1 week, the animals were randomly divided into a control group (n = 6) and crystal group (n = 6). Water and sterilized food were provided ad libitum. On the fifteenth day, the mice were killed and underwent heart perfusion. After rapid freezing in liquid nitrogen, the left kidneys were stored at −70°C. After fixation in 4% paraformaldehyde, the right kidneys were embedded in paraffin, sliced into 3‐μm sections and stained using a von Kossa kit (JieMei Gene) according to the manufacturer's instructions. Stained tissues were imaged under a light microscope (Zeiss AxioVision). Ten random fields from each kidney were used to calculate the percentage of crystal deposition area using Image‐Pro Plus 6.0 (Media Cybernetics) at ×200 magnification.

### 2.2 Cell culture and treatment

Human proximal tubular epithelial cells (HK‐2) were obtained from the ATCC and grown in DMEM/F12 (HyClone) containing 10% foetal bovine serum (FBS, Gibco), 100 U/mL penicillin and 100 µg/mL streptomycin in 5% carbon dioxide at a temperature of 37°C. COM crystals (Sigma‐Aldrich) were sterilized by heating overnight at 180°C as described previously.^15^ The crystals were then resuspended in DMEM/F12 without FBS using a magnetic stirrer at a suitable speed for 2 hours followed by immediate addition to HK‐2 cells for 48 hours. Lentiviruses containing HOXA1‐AS, small hairpin HOXA11‐AS and the negative control were synthesized by Obio Technology Corp., Ltd. The dual‐luciferase reporter plasmids were constructed by Genomeditech Co., Ltd. The mimics and inhibitors of miR‐124‐3p were provided by GenePharma Co., Ltd.

### 2.3 Quantitative real‐time polymerase chain reaction (qRT‐PCR)

Total RNA from HK‐2 cells or frozen left renal tissue was extracted with TRIzol and then reverse‐transcribed using a kit. Real‐time PCR was performed using the StepOnePlus™ RT‐PCR System (Applied Biosystems) with TaqMan SYBR Green. All reagents for qRT‐PCR were obtained from TaKaRa, Bio group and utilized per the manufacturer's protocol. The primer sequences are shown in Table S1. The expression of HOXA11‐AS, OPN, CD44 and MCP‐1 was normalized to which of β‐actin, and miR‐124‐3p was normalized to U6.

### 2.4 Cell proliferation assay (CCK‐8)

Cell proliferation was assessed using a cell counting kit‐8 (CCK‐8, Dojindo Laboratories) strictly according to the protocol supplied by the manufacturer. Briefly, HK‐2 cells were cultivated in 96‐well plates (8 × 10^3^ cells/well) and incubated overnight. Then, the medium was replaced with DMEM/F12 with or without COM (300 µg/mL) followed by incubation for 12, 24, 36 and 48 hours. Ten microlitres of the CCK‐8 reagent was added to each well and incubate for another 2 hours. The optical density (OD) of each well was measured at 450 nm to calculate the number of living cells using a multimode reader (BioTek). Three independent experiments were conducted in triplicate.

### 2.5 Cytotoxicity lactate dehydrogenase (LDH) assay

Lactate dehydrogenase is a stable cytoplasmic enzyme that is released once the cytomembrane ruptures. The activity of LDH was measured to represent the degree of cell damage using a Cytotoxicity LDH Assay Kit‐WST (Dojindo Laboratories). HK‐2 cells were cultivated in 96‐well plates (8 × 10^3^ cells/well) and incubated overnight. The medium was replaced with DMEM/F12 with or without COM (300 µg/mL), and the cells were cultivated for 48 hours. Then, HK‐2 cells were divided into three groups: (A) test substance group, cells treated with COM; (B) high control group, cells without COM but treatment for the subsequent cleavage of the wells (lysis buffer from the kit); (C) low control group, cells without any treatment. Each group was set background control wells, namely, cell‐free culture medium with or without COM wells. After incubation, the OD value of each well was measured at 490 nm using a multimode reader (BioTek). The actual OD value of each group was obtained from the measured OD of each group subtracting its background control OD. At last, the per cent cytotoxicity was calculated by the following equation. Cytotoxicity (%) = (A‐C)/(B‐C) × 100.

### 2.6 Cell apoptosis assay by flow cytometry

HK‐2 cells were seeded in 6‐well plates (1.5 × 10^6^ cells/well) and incubated for 24 hours. Next, the medium was replaced with DMEM/F12 with or without COM (300 µg/mL) and incubate for 48 hours. All cells in each well were collected and doubly stained with Annexin V‐FITC/propidium iodide (PI) or Annexin V‐APC/7‐ADD Apoptosis Detection kit (BD Biosciences) according to the manufacturer's instructions. Finally, the proportions of cell apoptosis were determined by flow cytometry (BD FACSCalibur). Three independent experiments were conducted in triplicate.

### 2.7 Western blot analysis

Total proteins were extracted from HK‐2 cells using RIPA lysis buffer (Beyotime, Haimen China) and separated by 12.5% sodium dodecyl sulphate‐polyacrylamide gel electrophoresis (SDS‐PAGE). The protein lysates were transferred onto a 0.22‐µm PVDF membrane (Millipore). After blocking in 5% nonfat milk at room temperature for 2 hours, the membranes were incubated with primary antibodies specific for CD44 (Abcam), OPN (Santa Cruz Biotechnology), MCP‐1 (Abcam) and β‐actin (Proteintech) at 4°C overnight. After three washes, the blots were incubated with IRDye 700/800‐conjugated secondary antibodies. The results were visualized and analysed using an Odyssey infrared scanner.

### 2.8 Enzyme‐linked immunosorbent assay (ELISA)

The levels of MCP‐1 in the cell culture supernatant were measured using a commercially available Chemiluminescent Immunoassay Kit (Cloud‐Clone) according to the manufacturer's instructions.

### 2.9 Dual‐luciferase reporter assay

HK‐2 cells were seeded in 6‐well plates (1.0 × 10^6^ cells/well) and incubated for 24 hours. Next, pGL3‐HOXA11‐AS WT, pGL3‐HOXA11‐AS MT, pGL3‐MCP‐1‐3‘UTR WT or pGL3‐MCP‐1‐3′UTR MT vectors were transfected in the HK‐2 cells in each well together with miR‐124‐3p or miR‐NC. After a 48‐hour incubation, the cells were harvested and subjected to the luciferase reporter assay using a Dual‐Luciferase Reporter Assay System (Promega) according to the manufacturer's instructions.

### 2.10 Statistical analysis

All data are presented as the mean ± SD, and the results were analysed by one‐way ANOVA for multigroup comparisons and Student's *t* test (two‐tailed) for differences between two groups using SPSS version 21.0 (SPSS, INC.). *P* < .05 indicated statistical significance.

## 3 RESULTS

### 3.1 HOXA11‐AS was up‐regulated in CaOx nephrolithiasis mouse kidney tissues and HK‐2 cells exposed to COM

**Figure 1:**
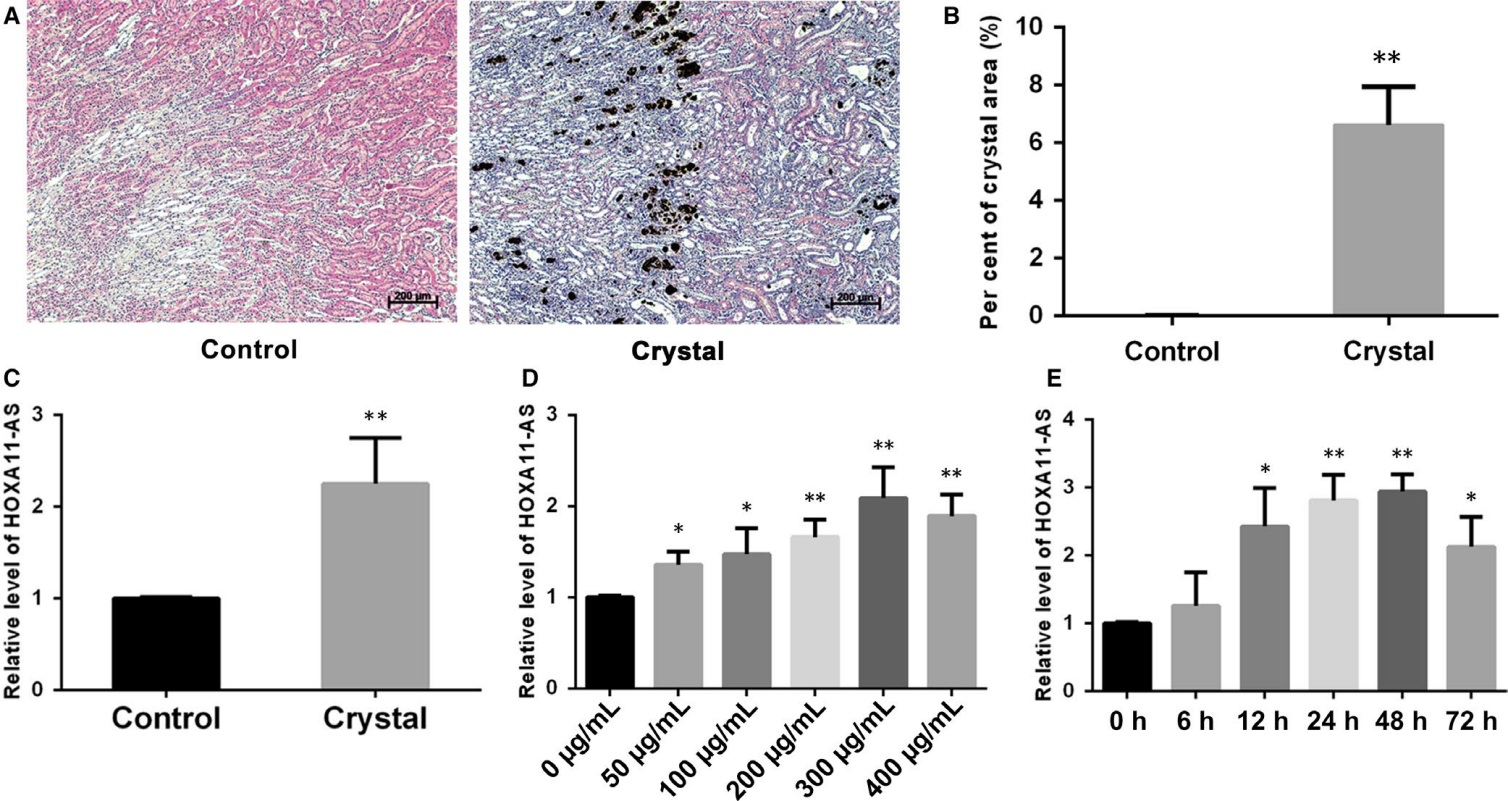
The expression of HOXA11‐AS in glyoxylate‐induced CaOx stone mouse kidney tissues and HK‐2 cells exposed to COM. A, CaOx crystal deposition in the tubules of the corticomedullary junction area in mouse kidneys was detected by von Kossa staining (Scale bar = 200 μm). B, Semiquantitative analysis of the positive crystal deposition per cent area obtained in 10 random views of each kidney at a magnification of 200 times. The expression of HOXA11‐AS in kidney tissues (C), HK‐2 cells exposed to different concentrations of COM for 48 h (D) and HK‐2 cells exposed to 300 µg/mL COM for various times (E). All values are expressed as the mean ± SD. ^*^ *P* < .5 vs control group, ^**^ *P* < .01 vs control group

To verify the microarray results, we established a glyoxylate‐induced CaOx stone mouse model and examined the expression of HOXA11‐AS in kidney tissues. The results showed significant CaOx crystal deposition by von Kossa staining (Figure 1A,B), and HOXA11‐AS was highly up‐regulated in the crystal group in mouse kidney tissues (Figure 1C). We also examined the expression of HOXA11‐AS in HK‐2 cells exposed to COM, an acknowledged cell model of CaOx nephrolithiasis. Our findings showed that HOXA11‐AS expression was clearly increased following the exposure of HK‐2 cells to COM, to a certain degree in a time‐ and dose‐dependent manner, reaching a peak at 300 µg/mL for 48 hours (Figure 1D,E). Consequently, we adopted this condition for the subsequent experiments.

### 3.2 HOXA11‐AS overexpression inhibited proliferation, promoted apoptosis and aggravated cellular damage in HK‐2 cells

**Figure 2:**
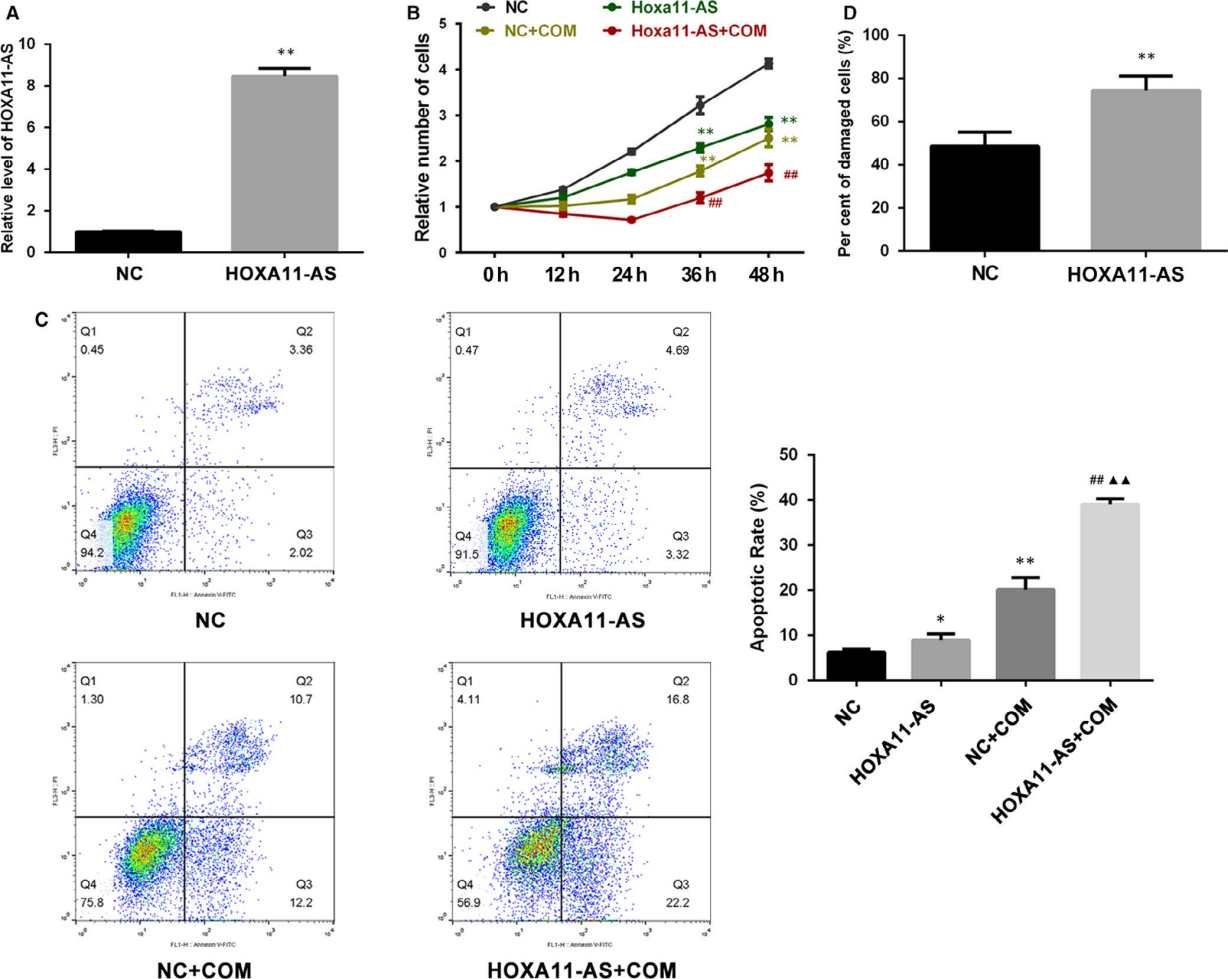
Functional effects of HOXA11‐AS overexpression in HK‐2 cells. A, HOXA11‐AS expression was validated by qRT‐PCR. B, The CCK‐8 assay showed that cell proliferation was inhibited by HOXA11‐AS overexpression with or without exposure to COM. C, Flow cytometry showed that cell apoptosis was promoted by HOXA11‐AS overexpression with or without exposure to COM. D, Cell damage was aggravated by HOXA11‐AS overexpression and assessed by measuring LDH activity. All values are expressed as the mean ± SD. ^*^ *P* < .5 vs NC group, ^**^ *P* < .01 vs NC group, ^##^ *P* < 0.01 vs NC + COM group, ^▲▲^ *P* < 0.01 vs HOXA11‐AS group

A stable HOXA11‐AS overexpression cell line was successfully constructed by lentiviral transfection, and the expression of HOXA11‐AS was validated by qRT‐PCR (Figure 2A). We investigated the proliferation of HK‐2 cells after HOXA11‐AS overexpression with cell counting kit‐8 (CCK‐8) and observed that HOXA11‐AS overexpression inhibited the proliferation of HK‐2 cells with or without exposure to COM (Figure 2B). Moreover, flow cytometry was utilized to determine the proportions of apoptotic cells and revealed a dramatic increase in cell apoptosis with or without exposure to COM after HOXA11‐AS up‐regulation (Figure 2C). In addition, the degree of cell damage was assessed by measuring LDH activity in the medium, and the results showed that HOXA11‐AS overexpression increased the cytotoxicity of COM to HK‐2 cells (Figure 2D).

### 3.3 HOXA11‐AS knockdown promoted proliferation, inhibited apoptosis and attenuated cellular injury in HK‐2 cells

**Figure 3:**
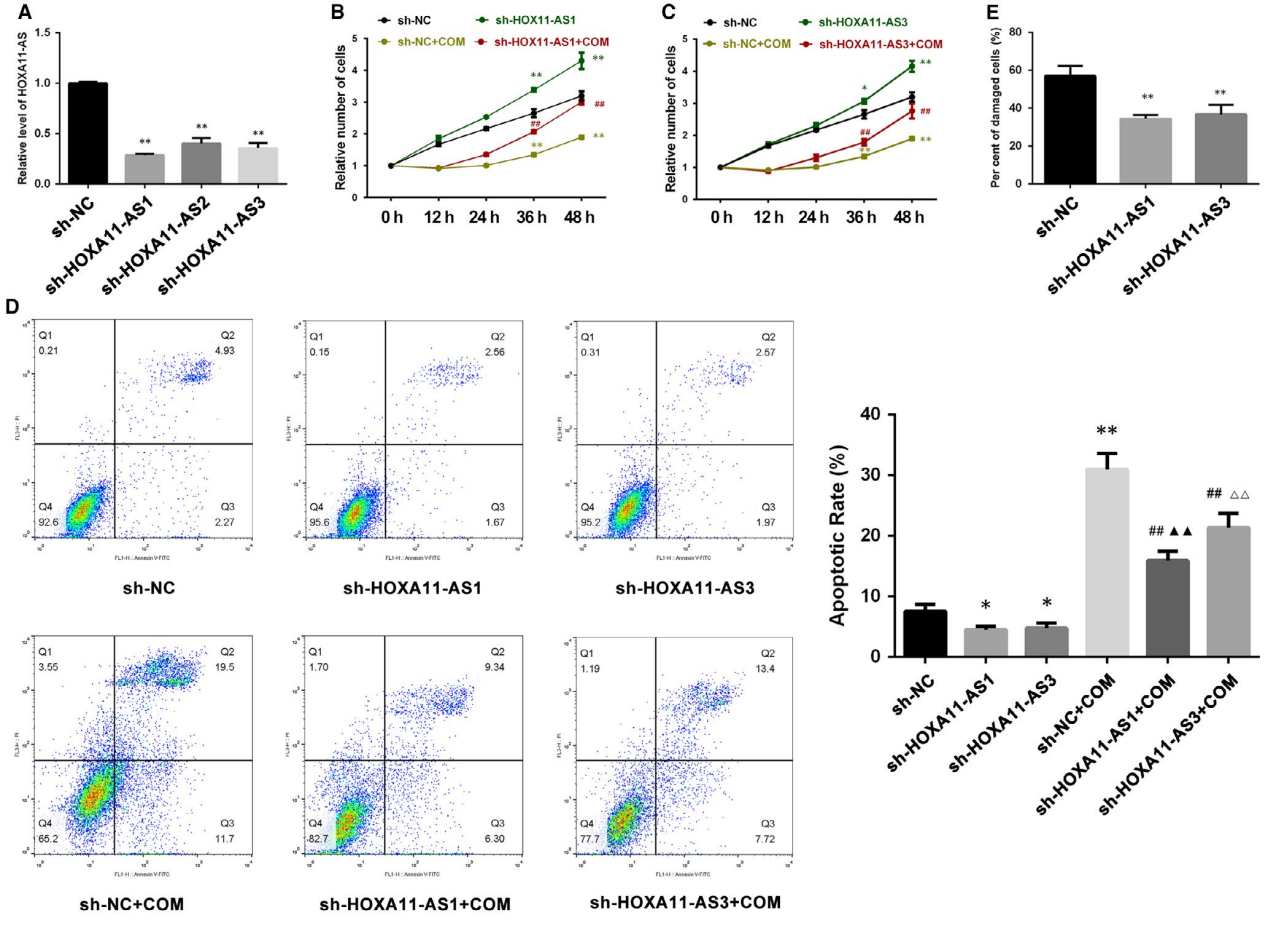
Functional effects of HOXA11‐AS knockdown in HK‐2 cells. A, HOXA11‐AS expression was validated by qRT‐PCR. B,C, The CCK‐8 assay showed that cell proliferation was promoted by HOXA11‐AS knockdown with or without exposure to COM. D, Flow cytometry showed that cell apoptosis was inhibited by HOXA11‐AS knockdown with or without exposure to COM. E, Cell damage was attenuated by HOXA11‐AS knockdown and assessed by measuring the LDH activity. All values are expressed as the mean ± SD. ^*^ *P* < .05 vs sh‐NC group, ^**^ *P* < .01 vs sh‐NC group, ^##^ *P* < .01 vs sh‐NC + COM group, ^▲▲^ *P* < 0.01 vs sh‐HOXA11‐AS1 group, ^△△^ *P* < 0.01 vs sh‐HOXA11‐AS3 group

We also constructed stable HOXA11‐AS knockdown cell lines using lentiviral small hairpin RNAs (sh‐HOXA11‐AS). The qRT‐PCR results showed that HOXA11‐AS expression was significantly down‐regulated by three small hairpin RNAs (Table 1), but the interference effects of sh‐HOXA11‐AS1 and sh‐HOXA11‐AS3 were much stronger than sh‐HOXA11‐AS2 (Figure 3A). Hence, we performed functional experiments using sh‐HOXA11‐AS1 and sh‐HOXA11‐AS3 cell lines. The results demonstrated than HOXA11‐AS knockdown promoted cell proliferation with or without COM exposure (Figure 3B,C), inhibited cell apoptosis (Figure 3D) with or without COM exposure and attenuated cell injury (Figure 3E) induced by COM, seemingly exhibiting a dose‐dependent relationship with degrees of interference.

**Table 1:** The sequences of siRNAs used in this study

Name	Primers 5′‐3′
sh‐HOXA11‐AS1	GCAGCATGCTTGTGCTCAA
sh‐HOXA11‐AS2	CCGATTTGCACGGTGACTT
sh‐HOXA11‐AS3	CCTGAGCCTTACGCTTCTT
sh‐NC	TTCTCCGAACGTGTCACGT

### 3.4 HOXA11‐AS regulated the expression of MCP‐1 in HK‐2 cells

**Figure 4:**
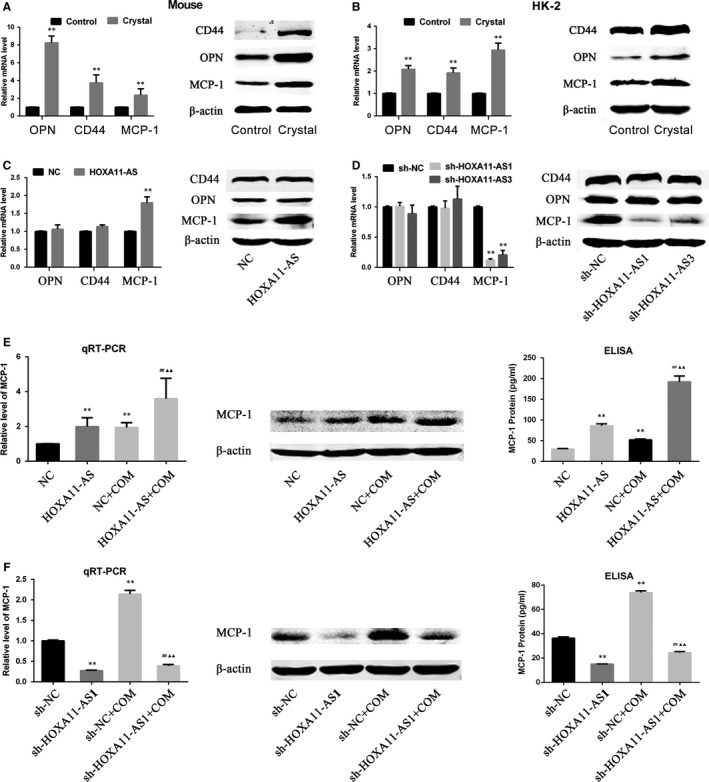
The regulatory effect of HOXA11‐AS on MCP‐1 expression. A, The expression levels of OPN, CD44 and MCP‐1 were measured by qRT‐PCR and Western blot analysis in the mouse kidneys. ^**^ *P* < .01 vs control group. B, The expression levels of OPN, CD44 and MCP‐1 were measured by qRT‐PCR and Western blot analysis in HK‐2 cells. ^**^ *P* < .01 vs control group. C, The expression levels of OPN, CD44 and MCP‐1 were measured by qRT‐PCR and Western blot analysis in the HOXA11‐AS overexpression cell line. ^**^ *P* < .01 vs NC group. D, The expression levels of OPN, CD44 and MCP‐1 were measured by qRT‐PCR and Western blot analysis in the sh‐HOXA11‐AS1 and sh‐HOXA11‐AS3 cell lines. ^**^ *P* < .01 vs sh‐NC group. E, The expression of MCP‐1 was examined in HOXA11‐AS overexpression cells exposed to COM by qRT‐PCR, Western blotting and ELISA. ^**^ *P* < .01 vs NC/sh‐NC group. ^**^ *P* < .01 vs NC group, ^##^ *P* < .01 vs HOXA11‐AS group, ^▲▲^ *P* < .01 vs NC + COM group. F, The expression of MCP‐1 was examined in sh‐HOXA11‐AS1 cells exposed to COM by qRT‐PCR, Western blotting and ELISA. ^**^ *P* < .01 vs sh‐NC group, ^##^ *P* < .01 vs sh‐HOXA11‐AS1 group, ^▲▲^ *P* < .01 vs sh‐NC + COM group. All values are expressed as the mean ± SD

In a previous study, the transcriptome of the animal model used in this study was investigated using a microarray technique, and the results showed that inflammation and immune reactivity through macrophage migration were involved in CaOx stone formation.^16^ In a comparison of the microarray analysis with three previous microarray reports (two reports concerning the ethylene glycol rat model and a COM crystal–exposed tubular cell model), the authors found serval genes that were commonly up‐regulated in all reports, among which OPN and MCP‐1 are well‐accepted stone‐related genes.^16^ Meanwhile, CD44, another stone‐related gene considered as the receptor for OPN, was also overexpressed in this microarray.^4^, ^16^ Moreover, in a recent microarray report using the ethylene glycol rat model, OPN, MCP‐1 and CD44 showed significantly up‐regulated expression.^17^ Therefore, we have been suggested that HOXA11‐AS might regulate the expression of these genes. Consequently, we first validated their expression in animal and cell models and, as expected, the results revealed their increased expression in vivo and in vitro. (Figure 4A,B). Next, their expression was measured in HOXA11‐AS knockdown or overexpression cell lines, and the results showed that the expression of MCP‐1 was increased in the HOXA11‐AS overexpression cell line and decreased in the HOXA11‐AS knockdown cell lines at the mRNA and protein levels, but the expression of OPN and CD44 was barely changed (Figure 4C,D). We further investigated the association between HOXA11‐AS level and MCP‐1 expression in the cell model using qRT‐PCR, WB and enzyme‐linked immunosorbent assay (ELISA). As expected, the results demonstrated that, regardless of exposure to COM, overexpressed HOXA11‐AS increased MCP‐1 expression in HK‐2 cells (Figure 4E), whereas HOXA11‐AS knockdown decreased MCP‐1 expression in HK‐2 cells (Figure 4F).

### 3.5 MiR‐124‐3p directly bound to HOXA11‐AS and the 3′UTR of MCP‐1 in HK‐2 cells

**Figure 5:**
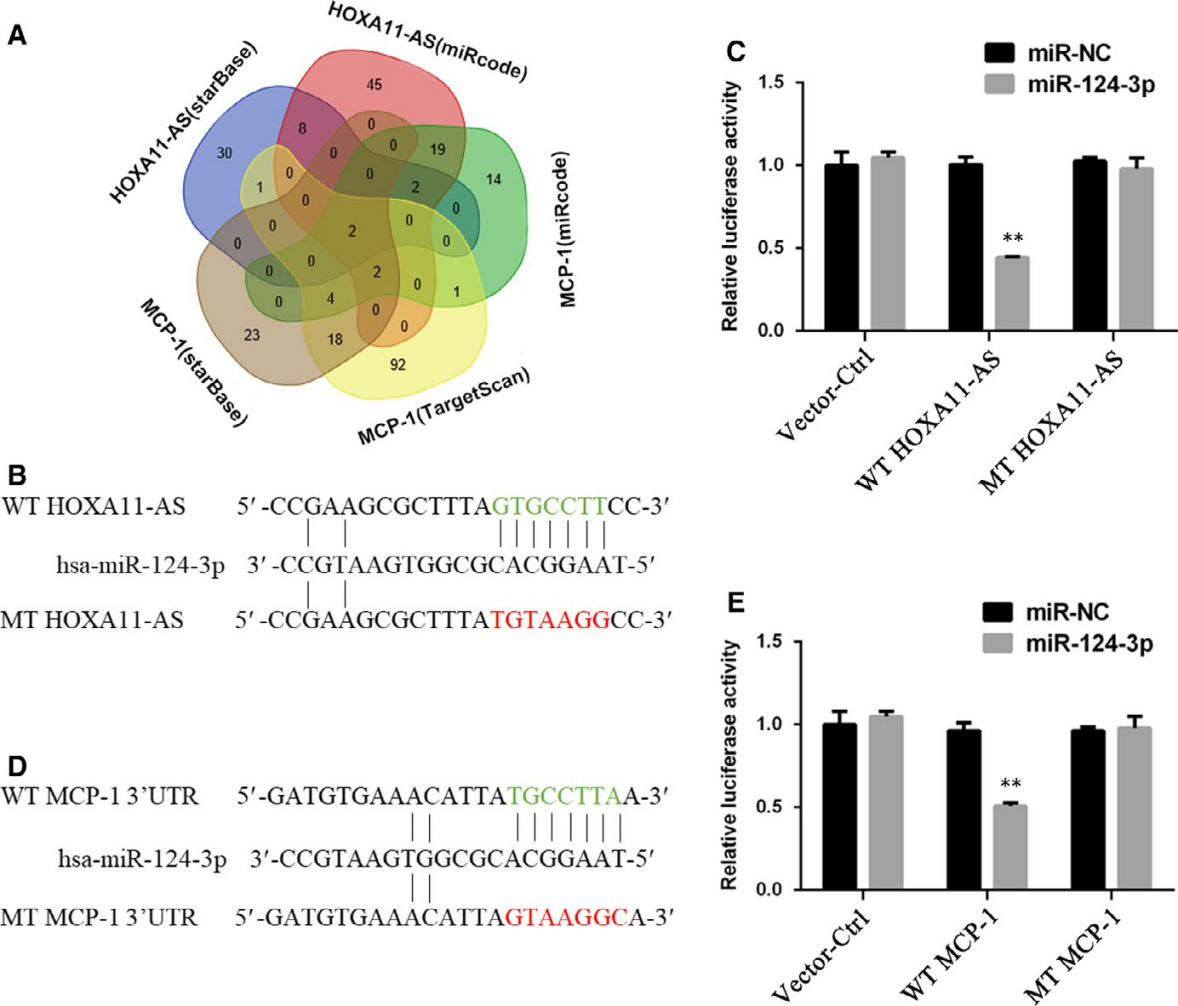
MiR‐124‐3p directly binds to HOXA11‐AS and the 3′UTR of MCP‐1. A, Venn diagram for miRNAs predicted to target HOXA11‐AS and MCP‐1 3′UTR by different online tools. B, The binding sites of miR‐124‐3p on HOXA11‐AS are shown in green, and the mutant sequence is shown in red. C, Dual‐luciferase reporter assays were performed using HK‐2 cells con‐transfected with control, WT HOXA11‐AS or MT HOXA11‐AS and miR‐124‐3p or miR‐NC for 48 h. D, The binding sites of miR‐124‐3p on HOXA11‐AS are shown in green, and the mutant sequence is shown in red. E, Dual‐luciferase reporter assays were performed using HK‐2 cells con‐transfected with control, WT MCP‐1 3′UTR or MT MCP‐1 3′UTR and miR‐124‐3p or miR‐NC for 48 h. All values are expressed as the mean ± SD. ^**^ *P* < .01 vs WT MCP‐1/ WT HOXA11‐AS group

According to the above‐described results, the expression level of MCP‐1 seemed to be positively related to HOXA11‐AS. Moreover, HOXA11‐AS has been shown to directly bind to AGO2 protein in a previous study.^10^ Therefore, we considered that HOXA11‐AS might function as a completing endogenous RNA (ceRNA) to regulate MCP‐1 expression. To verify this hypothesis, we predicted microRNAs that potentially bind to the MCP‐1 3′UTR or to HOXA11‐AS using several online tools, including starBase v3.0, TargetScan and miRcode 11, discovering that miR‐124‐3p and miR‐506‐3p could potentially target both (Figure 5A, Table S2). Next, using both microRNAs as keywords, we respectively searched the ceRNA researches related to either of them in PubMed and found that miR‐124‐3p had been previously reported to target HOXA11‐AS or MCP‐1. Furthermore, dual‐luciferase reporter assays were performed, and the results revealed that miR‐124‐3p overexpression largely reduced the luciferase activity of HK‐2 cells containing the HOXA11‐AS WT or MCP‐1 WT reporter gene, but barely suppressed the luciferase activity of HK‐2 cells with the HOXA11‐AS MT or MCP‐1 MT reporter gene (Figure 5B‐E). Consequently, miR‐124‐3p could directly bind to HOXA11‐AS and the 3′UTR of MCP‐1.

### 3.6 HOXA11‐AS modulated MCP‐1 expression through sponging miR‐124‐3p

**Figure 6:**
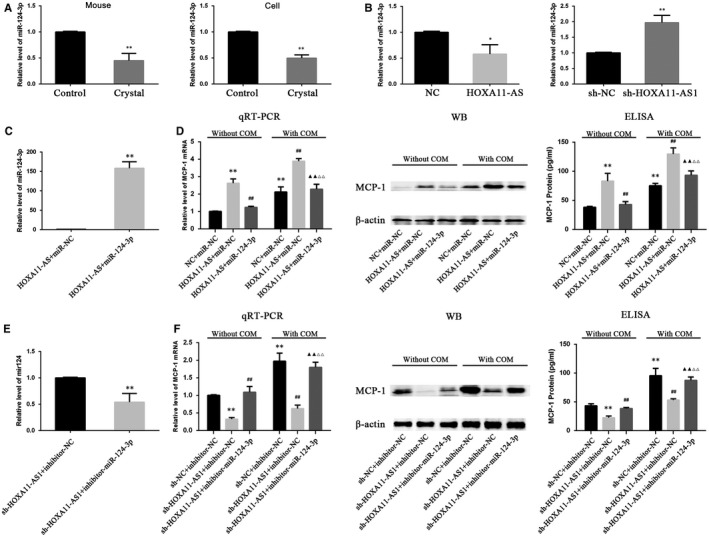
HOXA11‐AS modulates MCP‐1 expression through sponging miR‐124‐3p. A, The expression of miR‐124‐3p was measured in animals and a cell model by qRT‐PCR. ^**^ *P* < .01 vs control group. b, The expression of miR‐124‐3p was measured in HOXA11‐AS overexpression or knockdown cell lines by qRT‐PCR. ^*^ *P* < .05 vs NC group, ^**^ *P* < .01 vs sh‐NC group. C, The expression of miR‐124‐3p was measured by qRT‐PCR in HOXA11‐AS overexpression cells transfected with miR‐124‐3p mimics for 48 h. ^**^ *P* < .01 vs HOXA11‐AS group. D, The expression of MCP‐1 was measured by qRT‐PCR, WB and ELISA in HOXA11‐AS overexpression cells which were transfected with miR‐124‐3p or miR‐NC for 24 h followed by exposure to COM for 48 h. **P < .01 vs NC + miR‐NC group, ^##^ *P* < .01 vs HOXA11‐AS + miR‐NC group, ^▲▲^ *P* < .01 vs HOXA11‐AS + miR‐124‐3p group, ^△△^ *P* < .01 vs HOXA11‐AS + miR‐NC + COM group. E, The expression of miR‐124‐3p was measured by qRT‐PCR in sh‐HOXA11‐AS1 cells transfected with miR‐124‐3p inhibitors for 48 h. **P < .01 vs sh‐HOXA11‐AS1 group. F, The expression of MCP‐1 was measured by qRT‐PCR, WB and ELISA in sh‐HOXA11‐AS1 cells transfected with miR‐124‐3p inhibitors or inhibitor‐NC for 24 h followed by exposure to COM for 48 h. ^**^ *P* < .01 vs sh‐NC + inhibitor‐NC group, ##*P* < .01 vs. sh‐HOXA11‐AS1 + miR‐NC group, ^▲▲^ *P* < .01 vs sh‐HOXA11‐AS1 + inhibitor‐miR‐124‐3p group, ^△△^ *P* < .01 vs sh‐HOXA11‐AS1 + inhibitor‐NC + COM group. All values are expressed as the mean ± SD

The expression levels of miR‐124‐3p in mouse kidney and HK‐2 cells exposed to COM, as well as in HOXA11‐AS overexpression and knockdown cell lines, were measured by qRT‐PCR. The results demonstrated that miR‐124‐3p was down‐regulated in the animal and cell model (Figure 6A), and it was down‐regulated in the HOXA11‐AS overexpression cell line but up‐regulated in sh‐HOXA11‐AS1 cells (Figure 6B). This phenomenon in which miR‐124‐3p expression seemed to exhibit an opposite trend with HOXA11‐AS and MCP‐1 levels, coupled with its direct binding sites for them, led us consider that HOXA11‐AS could regulate MCP‐1 expression through sponging miR‐124‐3p. To validate this concept, we transfected miR‐124‐3p mimics into the constructed HOXA11‐AS overexpression cells and miR‐124‐3p inhibitors into the HOXA11‐AS knockdown cells. And 24 hours later, the cells were exposed to COM for 48 hours. As expected, the high‐level expression of MCP‐1 caused by HOXA11‐AS overexpression was markedly decreased by the miR‐124‐3p mimics at both mRNA and protein levels in HK‐2 cells with and without exposure to COM (Figure 6C,D), and equivalently, the miR‐124‐3p inhibitors significantly reversed the low‐level MCP‐1 expression in the sh‐HOXA11‐AS1 cell line (Figure 6E,F).

### 3.7 MiR‐124‐3p restored the inhibition of proliferation, promotion of apoptosis and aggravation of cellular damage induced by HOXA11‐AS overexpression

**Figure 7:**
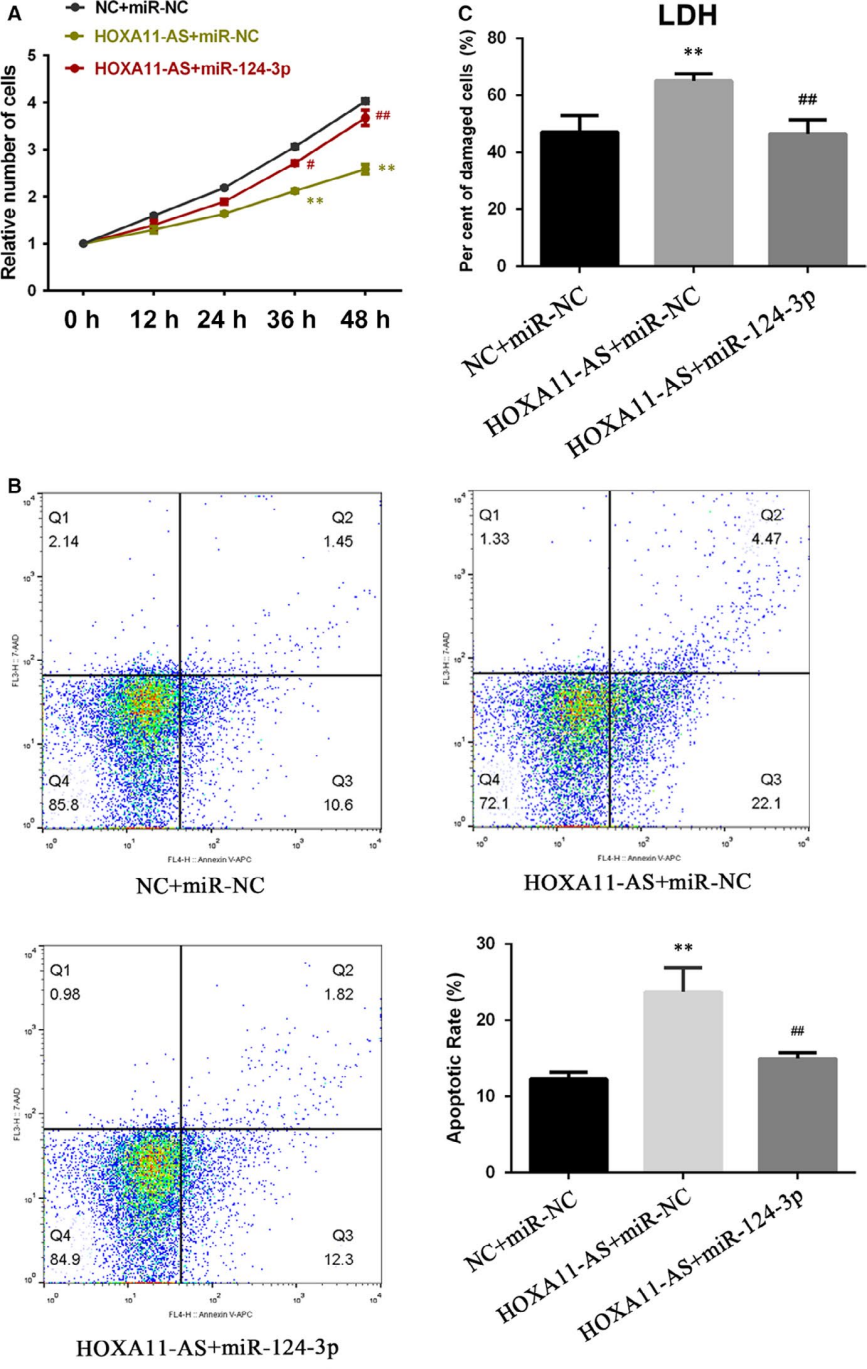
MiR‐124‐3p reverses HOXA11‐AS‐induced biological functions. A, The CCK‐8 assay showed that the inhibitory effect on proliferation of HOXA11‐AS overexpression was restored by miR‐124‐3p mimics. B, Flow cytometry showed that the apoptosis‐promoting effect of HOXA11‐AS was partially reversed by miR‐124‐3p. C, The LDH assay to examine cytotoxicity showed that the aggravation of cellular damage induced by overexpression of HOXA11‐AS was attenuated by miR‐124‐3p. All values are expressed as the mean ± SD. ^**^ *P* < .01 vs NC + miR‐NC group, ^##^ *P* < .01 vs HOXA11‐AS + miR‐NC group

To determine whether HOXA11‐AS exerts biological functions via sponging miR‐124‐3p, we transfected miR‐124‐3p mimics into HOXA11‐AS overexpressing cells and then measured the cellular proliferation, apoptosis and damage degree. As shown in Figure 7A, the CCK‐8 results illustrated that miR‐124‐3p partly restored the inhibitory effect of HOXA11‐AS on cell proliferation. Moreover, the flow cytometry results indicated that the COM‐induced promoting effect of HOXA111‐AS overexpression on apoptosis was partially reversed by miR‐124‐3p (Figure 7B). Furthermore, the cytotoxicity LDH assay showed that the aggravation of cellular damage induced by overexpression of HOXA11‐AS was attenuated by miR‐124‐3p transfection (Figure 7C).

## 4 DISCUSSION

It has been suggested that more than 70% of the human genome can be actively transcribed, but only 2% can be translated to protein; the rest are considered noncoding RNAs.^18^ Over the past decade, LncRNAs, which are members of the noncoding RNA family, have been well‐accepted as crucial biological RNAs in a wide variety of cancerous and noncancerous diseases rather than transcriptional ‘noise’ or cloning artefacts.^19^ In a previous study, we identified several CaOx stone‐related lncRNAs using a microarray technique and demonstrated that lncRNA CHCHD4P4 promoted renal EMT in CaOx nephrolithiasis.^8^ Another lncRNA, HOXA11‐AS, has been found to have multiple biological functions in a great deal of diseases in recent years.^10^, ^11^, ^12^, ^13^ It has been reported to serve as an oncogene in most cancers, such as non‐small‐cell lung cancer, gastric cancer, colorectal cancer and cervical cancer, among others.^20^ However, two studies have ascribed a role to HOXA11‐AS as a tumour suppressor gene in colorectal and epithelial ovarian cancer.^20^ In addition, HOXA11‐AS has been suggested to delay fracture healing by inhibiting proliferation and promoting apoptosis.^12^ Furthermore, in a recent research, HOXA11‐AS has been reported to promote proliferation, migration and inflammation‐related gene expression, thereby spurring inflammation in diabetic arteriosclerosis.^13^ The phenomenon in which HOXA11‐AS exhibits divergent functions in different diseases may be explained by tissue‐ and cell‐type‐specific lncRNAs.^14^ In the present study, the expression of HOXA11‐AS was significantly up‐regulated by CaOx crystals in vivo and in vitro (Figure 1), consistent with our previous microarray analysis. Functional experiments revealed that HOXA11‐AS inhibited proliferation, promoted apoptosis and aggravated cellular injury in CaOx nephrolithiasis (Figures 2 and 3).

To identify downstream genes of HOXA11‐AS, we compared a previous microarray analysis conducted to investigate CaOx stone‐related mRNAs using the same animal model^16^ with another recent microarray report from an ethylene glycol rat model^17^ and observed several commonly up‐regulated genes in these chips, among which, OPN, MCP‐1 and CD44 have been widely accepted to play key roles in CaOx stone formation. As a result, we first tested whether HOXA11‐AS could affect the expression of these three genes. As expected, their expression levels all increased in the mouse and cell models (Figure 4A,B). Only the change in MCP‐1 expression levels, but not OPN or CD44, kept pace with the levels of HOXA11‐AS in the reconstructed cell lines (Figure 4C,D), and this phenomenon was also observed when cells were exposed to COM (Figure 4E,F). Therefore, we suspected that HOXA11‐AS could regulate MCP‐1 expression in HK‐2 cells.

MCP‐1 expression shows a positive association with HOXA11‐AS, and HOXA11‐AS has been reported to directly bind AGO2 protein in a previous study.^10^ Therefore, we considered that HOXA11‐AS might function as a ceRNA to regulate MCP‐1 expression. First, we predicted microRNAs by online bioinformatics analysis and discovered that miR‐124‐3p and miR‐506‐3p could potentially target both MCP‐1 3’UTR and HOXA11‐AS. Subsequently, using these microRNAs as keywords, we respectively searched the research related to either of them in PubMed and found that only miR‐124‐3p had been previously reported to target HOXA11‐AS or MCP‐1. The expression of miR‐124‐3p was subsequently observed to be down‐regulated in kidney tissues and HK‐2 cells exposed to COM (Figure 6A,B). In addition, dual‐luciferase reporter assays validated that miR‐124‐3p directly bound to HOXA11‐AS and the 3′UTR of MCP‐1 (Figure 5). Furthermore, rescue assays revealed that miR‐124‐3p mimics could reduce the high‐level MCP‐1 expression at mRNA and protein levels caused by the artificial change of HOXA11‐AS in HK‐2 cells with and without exposure to COM, whereas the miR‐124‐3p inhibitors significantly restored the low‐level MCP‐1 expression (Figure 6C‐F). In summary, these results revealed that HOXA11‐AS could function as a ceRNA and modulated MCP‐1 expression through sponging miR‐124‐3p. By transfection of miR‐124‐3p mimics into HOXA11‐AS overexpression cells, we found that miR‐124‐3p could reverse the HOXA11‐AS overexpression‐induced biological functions, indicating that HOXA11‐AS exerted its biological functions via sponging miR‐124‐3p (Figure 7).

MCP‐1, the first discovered human CC chemokine, is a potent monocyte attractant,^21^ accounting for nearly 70%‐80% of the monocyte chemotactic activity in various types of renal inflammation.^3^ Under normal conditions, traces of MCP‐1 appear in renal epithelial, endothelial and mesangial cells. However, under pathological conditions such as exposure to COM, Ox or calcium ion, the expression of MCP‐1 is significantly elevated.^5^, ^22^, ^23^, ^24^, ^25^, ^26^ Moreover, MCP‐1 expression levels have been suggested to be greatly increased in urine specimens, stone‐adjacent tissues and the papillary tips of nephrolithiasis patients.^26^, ^27^, ^28^ Following exposure to COM or Ox, renal tubular epithelial cells generate massive amounts of reactive oxygen species (ROS) via the NADPH pathway, leading to cellular injury and apoptosis.^1^ The excessive ROS can then activate the NF‐κB, NLRP3, NACHT and LRR inflammatory pathways, leading to the secretion of many inflammatory factors such as OPN, MCP‐1, CD44, TGF‐β and IL‐1β, among others.^4^, ^5^ Successively, monocytes are chemotactically attracted by chemokines and converge to inflammatory sites where the monocyte can be activated to macrophages by COM and inflammatory cytokines and, thus, secrete more proinflammatory cytokines, followed by the enhancement of vasopermeability, recruitment of additional inflammatory cells, activation of complement and initiation of renal fibrosis.^6^, ^29^, ^30^, ^31^ Therefore, the inflammatory response is amplified by this amplificatory loop, thereby aggravating kidney damage, which is conducive to stone formation. It is well known that macrophages are phagocytic cells that can eliminate COM to some extent.^32^ However, activated macrophages have two major forms: the classically activated form (M1), which facilitates crystal formation and worsens the renal condition, and the alternatively activated form (M2), which suppresses crystal formation and exerts anti‐inflammatory and tissue healing effects.^32^ Furthermore, a recent study has suggested that CaOx promotes monocyte/macrophage differentiation into M1‐like macrophages. In summary, as the main chemokine for monocytes and the initial cytokines in the inflammatory cascade, MCP‐1 plays a vital role in CaOx stone formation. Additionally, proper mediation of the MCP‐1 level could be a good potential therapeutic target for renal nephrolithiasis.

In conclusion, we demonstrated that HOXA11‐AS was up‐regulated in CaOx nephrolithiasis both in vivo and in vitro. Moreover, HOXA11‐AS inhibited proliferation, promoted apoptosis and aggravated cellular damage in HK‐2 exposed to COM. We further revealed that HOXA11‐AS functioned as a ceRNA to inhibit proliferation, promote apoptosis, and aggravate cellular damage and modulate MCP‐1 expression through sponging miR‐124‐3p, thereby mediating CaOx crystal–related renal inflammation, and it may provide a good potential therapeutic target for renal nephrolithiasis.

## CONFLICTS OF INTEREST

The authors confirm that there are no conflicts of interest.

## AUTHOR CONTRIBUTIONS

Yinhui Li, Jihang Yuan and Shuhan Sun critically revised the manuscript. Yinhui Li, Jihang Yuan, Zhiyong Guo and Wei chen designed this study. Yinhui Li, Guiling Yan and Jie Zhang preformed the research and drafted the manuscript. Tao Ding, Minghan Li, Yiqing zhu and Yupeng Yin performed the acquisition, analysis and interpretation of the data; Zhiyong Guo revised the manuscript critically and finally approved the version to be published. All authors have read and approved the final manuscript.
