# Supplementary figures and images for: LncRNA HOXA11‐AS regulates calcium oxalate crystal–induced renal inflammation via miR‐124‐3p/MCP‐1
Source: J Cell Mol Med. 2019 Nov 3;24(1):238–49. doi: 10.1111/jcmm.14706 (PMC6933336; doi:10.1111/jcmm.14706)

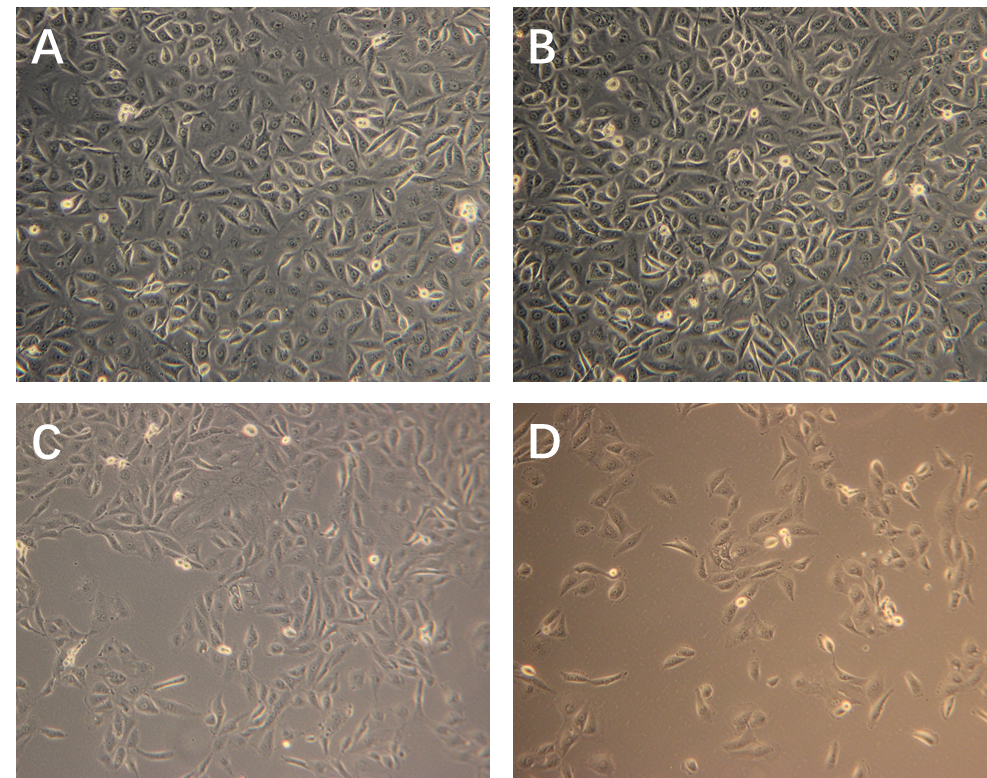

Supplement: Supplementary file 1 [file JCMM-24-238-s001.tif]
