# Supplementary material for: LncRNA HOXA11‐AS regulates calcium oxalate crystal–induced renal inflammation via miR‐124‐3p/MCP‐1
Source: J Cell Mol Med. 2019 Nov 3;24(1):238–49. doi: 10.1111/jcmm.14706 (PMC6933336; doi:10.1111/jcmm.14706)
Supplement: Supplementary file 2 [file JCMM-24-238-s002.docx]

**TABLE S1** The sequences of primers used for qRT-PCR

| **Name** | **Primers 5’-3'** |
| --- | --- |
| β-actin(human)-F  β-actin(human)-R  HOXA11-AS (human)-F  HOXA11-AS (human)-R  MCP-1 (human)-F  MCP-1 (human)-R  OPN (human)-F  OPN (human)-R  CD44(human)-F  CD44(human)-R  β-actin(mouse)-F  β-actin(mouse)-R  HOXA11-AS (mouse)-F  HOXA11-AS (mouse)-R  MCP-1 (mouse)-F  MCP-1 (mouse)-R  OPN (mouse)-F  OPN (mouse)-R  CD44(mouse)-F  CD44(mouse)-R  U6-F  U6-R  U6-RT  miR-124-3p-F  miR-124-3p-R  miR-124-3p-RT | GGGAAATCGTGCGTGACATTAAG  TGTGTTGGCGTACAGGTCTTTG  CGGCTAACAAGGAGATTTGG  AGGCTCAGGGATGGTAGTCC  GATCTCAGTGCAGAGGCTCG  TTTGCTTGTCCAGGTGGTCC  CTCCATTGACTCGAACGACTC  CAGGTCTGCGAAACTTCTTAGAT  CTGCCGCTTTGCAGGTGTA  CATTGTGGGCAAGGTGCTATT  CTGTCCACCTTCCAGCAGATGT  GTCGCCTTCACCGTTCCAGTT  GCTCTCATTCACGGTCACTTC  TCTGGCTCTGAGGAGTCACT  TAAAAACCTGGATCGGAACCAAA  GCATTAGCTTCAGATTTACGGGT  ATCTCACCATTCGGATGAGTCT  TGTAGGGACGATTGGAGTGAAA  AGCGGCAGGTTACATTCAAA  CAAGTTTTGGTGGCACACAG  GCTTCGGCAGCACATATACTAAAAT  CGCTTCACGAATTTGCGTGTCAT  CGAGCACAGAATCGCTTCACGAATTTGCGTGTCAT  CGAATAAGGCACGCGGTGAA  GTGCAGGGTCCGAGGTATTC  GTCGTATCCAGTGCAGGGTCCGAGGTATTCGCACTGGATACGACTTGGCA |

**TABLE S2 Detail data for venn diagram of Figure 5A**

| **Intersection** | **NO.** | **miRNAs** |
| --- | --- | --- |
| **HOXA11-AS(miRcode) HOXA11-AS(starBase) MCP-1(TargetScan) MCP-1(miRcode)**  **MCP-1(starBase)** | 2 | miR-124-3p miR-506-3p |
| **HOXA11-AS(miRcode) MCP-1(TargetScan) MCP-1(miRcode)**  **MCP-1(starBase)** | 2 | miR-33b-5p miR-33a-5p |
| **HOXA11-AS(miRcode) HOXA11-AS(starBase) MCP-1(miRcode)** | 2 | miR-3619-5p miR-761 |
| **MCP-1(TargetScan) MCP-1(miRcode)**  **MCP-1(starBase)** | 4 | miR-22-3p miR-206 miR-613 miR-23b-3p |
| **HOXA11-AS(miRcode) HOXA11-AS(starBase)** | 8 | miR-301b-3p miR-24-3p miR-4295 miR-208b-3p miR-3666 miR-148b-3p miR-148a-3p miR-208a-3p |
| **HOXA11-AS(starBase) MCP-1(TargetScan)** | 1 | miR-2355-5p |
| **HOXA11-AS(miRcode) MCP-1(miRcode)** | 19 | miR-1297 miR-26a-5p miR-16a miR-1907 miR-15c miR-322 miR-497 miR-15a miR-16c miR-33-5p miR-16 miR-124b miR-15b miR-214 miR-195 miR-424 miR-16b miR-4465 miR-26b-5p |
| **MCP-1(TargetScan) MCP-1(miRcode)** | 1 | miR-23c |
| **MCP-1(TargetScan) MCP-1(starBase)** | 18 | miR-1-3p miR-23a-3p miR-369-3p miR-624-5p miR-335-5p miR-548o-3p miR-409-3p miR-323a-3p miR-577 miR-374a-5p miR-374b-5p miR-524-5p miR-520d-5p miR-579-3p miR-4766-3p miR-495-3p miR-543 miR-1323 |
| **HOXA11-AS(starBase)** | 30 | let-7f-5p let-7i-5p miR-518a-3p let-7a-5p let-7e-5p let-7b-5p miR-515-5p miR-130b-3p let-7d-5p miR-518f-3p let-7c-5p miR-518c-3p miR-4458 miR-605-3p miR-3145-3p miR-518d-3p miR-152-3p miR-1193 miR-454-3p let-7g-5p miR-301a-3p miR-98-5p miR-7853-5p miR-214-3p miR-130a-3p miR-223-3p miR-519e-5p miR-518b miR-105-5p miR-4500 |
| **HOXA11-AS(miRcode)** | 45 | miR-129-5p miR-130c miR-670 miR-183 miR-125b-5p miR-129b-5p miR-205b miR-146b-5p miR-19b miR-351 miR-190b miR-29b miR-721 miR-199a-5p miR-29c miR-205a mir-4500 miR-24b miR-129a-5p miR-24a mir-4458 miR-190a miR-223 mir-98 miR-145 miR-19a miR-24 miR-146a miR-454 miR-190 miR-205 miR-208a miR-152 miR-301b miR-29d miR-125a-5p miR-301a miR-4319 miR-184 let-7 miR-199b-5p miR-130a miR-146c miR-208b miR-29a |
| **MCP-1(miRcode)** | 14 | miR-122 miR-122a miR-9a miR-1a miR-9 miR-1352 miR-23a miR-138a miR-9b miR-22 miR-1b miR-138b miR-23b miR-138 |
| **MCP-1(TargetScan)** | 92 | miR-4318 miR-3667-5p miR-6074 miR-3691-3p miR-6507-5p miR-496.2 miR-4650-3p miR-4528 miR-637 miR-616-5p miR-10b-3p miR-95-5p miR-593-3p miR-3942-5p miR-4667-5p let-7c-3p miR-3184-3p miR-4704-5p miR-8089 miR-548aw miR-3679-3p miR-548e-5p miR-3132 miR-493-5p miR-130a-5p miR-371b-5p miR-3923 miR-197-5p miR-5696 miR-4742-3p miR-633 miR-1231 miR-4703-5p miR-7153-3p miR-635 miR-3908 miR-6824-5p miR-6793-3p miR-6783-5p miR-3125 miR-374b-3p miR-6859-5p miR-3714 miR-302c-5p miR-33a-3p miR-1915-3p miR-3149 miR-664a-3p miR-6840-3p miR-5692c miR-5002-5p miR-1273g-5p miR-4725-3p miR-6844 miR-5692a miR-4271 miR-7155-5p miR-4700-5p miR-3153 miR-518a-5p miR-5688 miR-6505-5p miR-1277-5p miR-527 miR-6739-5p miR-586 miR-5700 miR-6780b-5p miR-4668-5p miR-3910 let-7g-3p miR-2054 miR-6774-5p miR-3163 miR-2110 miR-6733-5p miR-3692-3p miR-580-5p miR-4760-3p miR-3158-5p miR-373-5p miR-4726-3p miR-664b-3p miR-5692b miR-7159-5p let-7a-2-3p miR-3065-5p miR-3916 miR-203a-5p miR-6764-5p miR-3143 miR-6124 |
| **MCP-1(starBase)** | 23 | miR-320b miR-141-3p miR-325 miR-4756-5p miR-491-5p miR-588 miR-320c miR-4739 miR-212-5p miR-3187-3p miR-379-5p miR-421 miR-1321 miR-3150a-3p miR-200a-3p miR-488-3p miR-4701-5p miR-371a-5p miR-498 miR-320a miR-320d miR-766-5p miR-889-3p |
